# Genetic Insights into Obesity and Brain: Combine Mendelian Randomization Study and Gene Expression Analysis

**DOI:** 10.3390/brainsci13060892

**Published:** 2023-05-31

**Authors:** Leian Chen, Shaokun Zhao, Yuye Wang, Xiaoqian Niu, Bin Zhang, Xin Li, Dantao Peng

**Affiliations:** 1 Department of Neurology, China-Japan Friendship Hospital (Institute of Clinical Medical Sciences), Chinese Academy of Medical Sciences & Peking Union Medical College, Beijing 100029, China; 2 State Key Laboratory of Cognitive Neuroscience and Learning, Beijing Normal University, Beijing 100875, China; 3 Department of Neurology, Peking University China-Japan Friendship School of Clinical Medicine, Beijing 100029, China

**Keywords:** obesity, body mass index, cerebral cortex, Mendelian randomization analysis, gene expression, cognitive impairment

## Abstract

As a major public-health concern, obesity is imposing an increasing social burden around the world. The link between obesity and brain-health problems has been reported, but controversy remains. To investigate the relationship among obesity, brain-structure changes and diseases, a two-stage analysis was performed. At first, we used the Mendelian-randomization (MR) approach to identify the causal relationship between obesity and cerebral structure. Obesity-related data were retrieved from the Genetic Investigation of ANthropometric Traits (GIANT) consortium and the UK Biobank, whereas the cortical morphological data were from the Enhancing NeuroImaging Genetics through Meta-Analysis (ENIGMA) consortium. Further, we extracted region-specific expressed genes according to the Allen Human Brian Atlas (AHBA) and carried out a series of bioinformatics analyses to find the potential mechanism of obesity and diseases. In the univariable MR, a higher body mass index (BMI) or larger visceral adipose tissue (VAT) was associated with a smaller global cortical thickness (*p*_BMI_ = 0.006, *p*_VAT_ = 1.34 × 10^−4^). Regional associations were found between obesity and specific gyrus regions, mainly in the fusiform gyrus and inferior parietal gyrus. Multivariable MR results showed that a greater body fat percentage was linked to a smaller fusiform-gyrus thickness (*p* = 0.029) and precuneus surface area (*p* = 0.035). As for the gene analysis, region-related genes were enriched to several neurobiological processes, such as compound transport, neuropeptide-signaling pathway, and neuroactive ligand–receptor interaction. These genes contained a strong relationship with some neuropsychiatric diseases, such as Alzheimer’s disease, epilepsy, and other disorders. Our results reveal a causal relationship between obesity and brain abnormalities and suggest a pathway from obesity to brain-structure abnormalities to neuropsychiatric diseases.

## 1. Introduction

Obesity remains a major public-health challenge due to its rising prevalence [1]. It is well documented that obesity is associated with an increased risk of mortality as well as numerous health disorders, including diabetes, hypertension, cardiovascular diseases, metabolic syndromes, and even cancer [2]. The effect of obesity on the central nervous system is receiving growing wide attention. Obesity impairs cognitive function, manifesting deficits in episodic memory, selective attention, executive functions, decision-making processing, and other cognitive domains [3]. Regarding obesity as a risk factor for dementia, several international initiatives have adopted weight management as a dementia-prevention measure [4]. Neuroimaging studies have reported brain-structural alterations in obese individuals [5,6,7,8]. Recent evidence has documented that obesity is linked to decreased gray-matter volume or density at both global and regional levels [3]. There was a significant correlation between elevated body mass index (BMI) and cortical atrophy in the prefrontal, frontal, temporal, and occipital cortex [9]. However, this association was different in other studies [9,10]. A higher volume of intra-abdominal fat is associated with greater cortical thickness, including the cingulate, fusiform, and insular cortex [11,12,13]. All results are based on observational studies, but observational associations are not able to discriminate between correlations and causal relationships. More importantly, reverse causation and residual confounders may bias the results, leading to distortion of the true relationship. For example, impairments in regions responsible for energy intake or eating behavior make people prone to aberrant overeating, thus contributing to obesity [9]. Therefore, the relationship between obesity and brain structure remains unclear. 

At the same time, little is known about the underlying mechanisms if obesity does affect the brain. Previous studies have revealed the shared genetics between obesity and neuropsychiatric disorders [14]. Obesity influences the transcriptional activity of the brain genes [15,16,17]. Great progress has been made in this field, but too little work has been devoted to linking obesity to gray matter and genetic information in the human brain. If obesity does affect certain brain structures, would there be changes in gene expression in those affected brain regions, and would these gene changes be associated with certain diseases? The brain-wide gene-expression atlases have connected spatial variations in gene expression to brain structure and function [18], which will be helpful to offer a clearer understanding of the association between obesity and brain structure.

Mendelian randomization (MR) is a statistical approach that uses genetic variants, such as single-nucleotide polymorphisms (SNPs), as instrumental variables to infer causality between exposure and diseases [19]. The alleles of a given SNP are randomly allocated to individuals during human-gamete formation, which happens before any exposure or outcome, so inherited variants are independent of potentially confounding environmental exposure [20]. A common strategy used to study the human brain is neuroimaging techniques, mainly magnetic resonance imaging (MRI). Nevertheless, there is no previous research using the Mendelian randomization approach to makes up for the problem of obesity and the human brain. In the present research, the study set out to determine the causal associations between obesity and the cerebral cortex using the MR approach. Using the data from public sources, body mass index (BMI), waist-to-hip ratio (WHR), body fat percentage (BFP), and visceral adipose tissue (VAT) were included in our study to comprehensively investigate their relationships with the brain. Subsequently, we acquired gene expression in obesity-related brain regions and tested which biological processes or functional pathways were linked to obesity-related brain abnormalities. By analyzing the relevance of obesity and diseases from the perspective of gene expression, our study will provide a deeper insight into the effects of obesity on the brain.

## 2. Materials and Methods

### 2.1. Study Design

**Figure 1:**
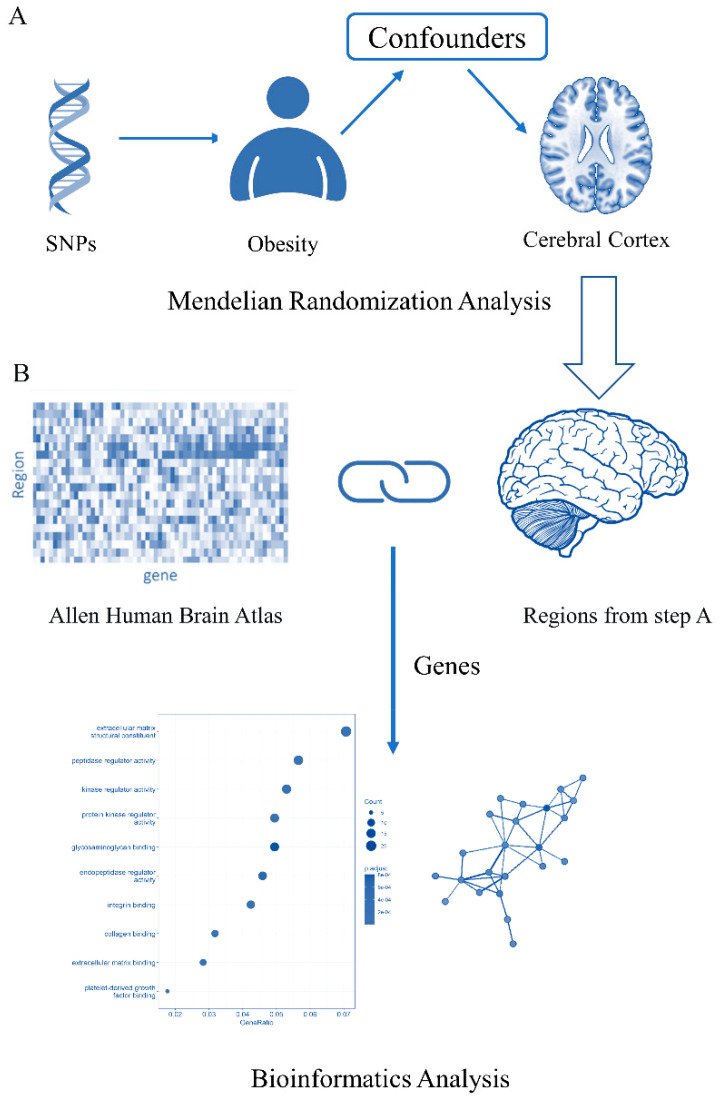
Flow diagram of the analysis pipeline used in the study. (**A**). Two-sample MR analyses were performed to evaluate the causal effect of obesity on the cortex structure. (**B**). Bioinformatics analyses were performed using genes that were differentially expressed in brain regions significantly indicated in step A.

The workflow of this study is summarized in Figure 1. In the first stage, we performed univariate two-sample MR analyses to evaluate the causal effect of obesity on the cortex structure. Then, based on the result of the two-sample MR analysis, we conducted additional multivariate MR (MVMR) analyses to further investigate the impact of obesity on the cortex. In the second stage, we extracted the specific expression genes of the brain regions that were statistically significant in MR analyses from the Allen Human Brain Atlas (https://human.brain-map.org/, accessed on 30 December 2022). We used these genes to conduct several bioinformatics analyses, including enrichment analysis for functional or disease characteristics and protein–protein interactions on the STRING website. MR analysis was performed based on the recommendations of the STROBE-MR statement [21]. It should fulfill three core assumptions: (1) The instrumental variables (IVs) are associated with the exposure, (2) IVs are not associated with confounders, and (3) IVs influence the outcome only via the given exposure [20].

### 2.2. Data Sources for Obesity and Cortical Structure Phenotype

Four obesity-related traits, including BMI, WHR, BFP, and VAT, were considered as the exposure factors in our analysis. The summary-level genome-wide association study (GWAS) data correlated with BMI were obtained from a meta-analysis of GWASs of European-ancestry participants from the GIANT consortium and the UK Biobank [22]. This meta-analysis of GWASs of BMI included nearly 700,000 individuals. Genetic variants associated with waist-to-hip ratio (WHR) were also based on a large-scale GWAS (697,734 individuals) from the GIANT consortium (*n* = 212,248) and the UK Biobank (*n* = 485,486) [23]. We selected SNPs associated with body fat percentage (BFP) and visceral adipose tissue (VAT) from the UK Biobank [24]. The number of samples and other details are shown in Table 1.

The human cortical structure-related GWAS data were obtained from the Enhancing NeuroImaging Genetics through Meta-Analysis (ENIGMA) Consortium [25], including 23,909 participants of European descent from 49 cohorts. The biochemical GWASs, excluding UK Biobank GWASs, were used to eliminate statistical inflation arising from sample overlap between the ENIGMA and UK Biobank cohorts. The measured cerebral phenotype included cortical thickness (TH) and cortical surface area (SA). The cerebral cortex was divided into 34 brain regions based on the Desikan–Killiany cortical atlas [26]. The regions were averaged between both hemispheres.

### 2.3. Selection of Genetic Variants

We used single nucleotide polymorphisms (SNPs) as instrumental variables. To identify the causal relationship between obesity and the human cerebral cortex, we used four indicating different aspects of obesity, such as adipose proportion or distribution. The threshold of genome-wide significance for SNPs was set at *p* < 5 × 10^−8^. To obtain independent instrumental variables, all significant SNPs were clumped according to linkage disequilibrium (r^2^ < 0.001 with ±10,000 kb based on the 1000 Genomes Project identified therein as having European ancestry). Then, we harmonized all SNPs to ensure that effect estimates corresponded to the same allele. The proportion of variance explained by each SNP was calculated via the formula *R*^2^ = 2 × β^2^ × MAF × (1 − MAF). β is the estimate of the effect of SNP on exposure, and MAF is minor-allele frequency. In addition, F-statistics were also calculated to avoid bias due to weak IVs [27]. An SNP with F-statistics less than 10 was defined as a weak instrumental variable [28], and all weak IVs were removed. F-statistics for each exposure were calculated as follows (Equation (1)) [29]:

(1) $$F=\frac{N-K-1}{K}\frac{R^{2}}{1-R^{2}}$$

where *K* means the number of SNPs, *N* means the number of the sample size, and *R*^2^ is the proportion of variance explained by all SNPs. In addition, we also removed SNPs associated with other potential risk factors based on the results in the PhenoScanner (www.phenoscanner.medschl.cam.ac.uk, accessed on 12 December 2022). MR Pleiotropy RESidual Sum and Outlier (MR-PRESSO) was applied to remove the underlying outliers (based on 10,000 simulations) [30]. Table 1 shows the basic characteristics of exposure factors.

### 2.4. Mendelian Randomization 

Two-sample Mendelian randomization (MR) was employed to further explore and quantify the impact of obesity on cortex structure. Inverse-variance weighting (IVW), weight median (WM), and MR-Egger were used to estimate the causal relationship between exposure (obesity) and outcome (brain structure). Given the presence of heterogeneity among SNPs, random-effect IVW was used as the primary method, in which the slope of the weighted regression represented the resulting estimate [31]. In sensitivity analyses, we evaluated heterogeneity by IVW and MR-Egger. Cochran’s Q test (*p* < 0.05 indicates heterogeneity) was adopted to assess the heterogeneity among SNPs in IVW estimates [32]. We also conducted a leave-one-out analysis to estimate the influence of outlying or pleiotropic genetic variants. The potential pleiotropy of these SNPs was evaluated via MR-Egger regression intercept [33]. MR Steiger analysis was performed to estimate the potential reverse-causal impact of various obesity traits on each brain phenotype [34]. A two-sided *p*-value < 0.05 was regarded as statistically significant. For region-level analyses, there were 34 components. Therefore, a Bonferroni-corrected *p*-value < 0.05/34 (1.47 × 10^−3^) was taken to indicate statistical significance for a causal association, whereas a *p* < 0.05 was regarded as a potential, yet to be confirmed, causal association. The sample size and the power were calculated via a web-based tool (mRnd: Power calculations for Mendelian Randomization, https://shiny.cnsgenomics.com/mRnd/, accessed on 12 December 2022) [35]. We estimated sample sizes at power 80% and alpha 0.05.

As an extension of MR, multivariable MR (MVMR) uses genetic variants associated with multiple potentially related exposures to estimate the effect of each exposure on a single outcome [36]. In our study, multivariable MR analysis was an essential supplementary strategy to further assess the causal effects of obesity on cerebral-cortex traits. According to the results of two-sample MR analyses, we focused on the association between obesity indexes and the statistically significant brain cerebral cortex. We used regression-based IVW for MVMR. The SNPs utilized in MVMR were IV combinations of each exposure factor. We restricted the analysis to SNPs that were clumped on r^2^ < 0.01 with ±10,000 kb. All analyses were performed using the packages “TwoSampleMR” (version 0.5.6), “MendelianRandomization” (version 0.6.0), and “MRPRESSO” (version 1.0) in R (version 4.1.2). The “ggplot2” and “ggseg” packages in R were used to create plots.

### 2.5. Genetic Associations with Brain-Imaging Measurement

We analyzed the human-microarray dataset from the Allen Human Brain Atlas (AHBA) (https://human.brain-map.org/, accessed on 30 December 2022). The AHBA is a free atlas, integrating structure, function, and gene-expression database [37]. The previous MR analyses showed which brain regions were affected by obesity, and these regions were considered vulnerable regions in our research. By using the “differential search function” provided by the AHBA, we downloaded genes that showed enhanced expression in those vulnerable regions compared to the whole cerebral cortex [38]. Genes were considered differentially expressed within each region if they had a *p*-value of <0.05 and an absolute log-fold change of >2. Differentially expressed genes of obesity-related predicted by MR-analysis regions were used to perform enrichment analysis in the Kyoto Encyclopedia of Genes and Genomes (KEGG) pathways, Gene Ontology (GO) terms, and Disease Ontology (DO). FDR correction for the KEGG, GO, and DO was used simultaneously to determine the significance using an adjusted *p*-value cut-off of *p* < 0.05. Those gene-expression experiments were carried out in R version 4.1.2. Then, those genes were uploaded to the STRING website (https://cn.string-db.org/, accessed on 30 December 2022) to draw protein–protein interaction (PPI) network maps. Because PPI-network analysis is primarily concerned with the biological processes at both a molecular and a systems level, the focus of GO in this study was on the biological processes (BPs), and the results of cellular components and molecular functions can be seen in the File S3 (Figures S1 and S2).

## 3. Results

### 3.1. Causal Association of Obesity with Cerebral Cortex

**Figure 2:**
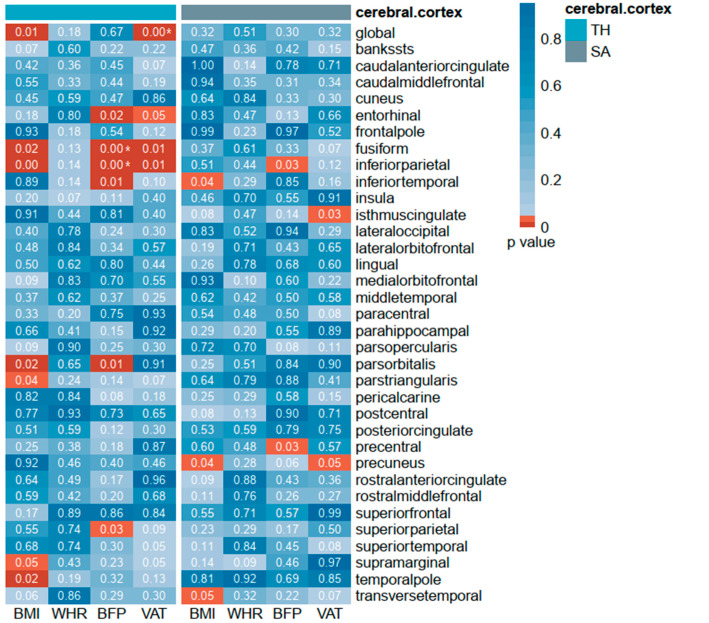
IVW estimates of various obesity parameters on brain cortical thickness and surface area. IVW estimates of various obesity parameters (BMI, WHR, BFP, and VAT) on brain cortical thickness and surface area. The color of each block represents the IVW-derived *p*-values of every MR analysis. The *p*-values < 0.05 are shown in red squares and *p*-values > 0.05 are shown in white or blue squares. BMI: body mass index; WHR: waist–hip ratio; BFP: body fat percentage; VAT: visceral adipose tissue; SA: surface area; TH: thickness; * *p*-value < 0.01.

**Figure 3:**
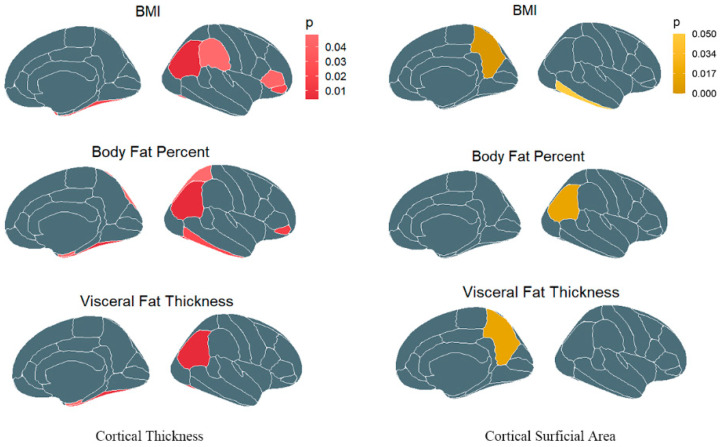
The obesity-related regions from the perspective of different measurements. The red brain regions represent the regions where cortical thickness is affected in our MR study, whereas the yellow represents regions where the cortical surface area is affected.

In total, 513 index SNPs were selected to genetically predict BMI, 72 index SNPs for WHR, 300 index SNPs for body fat percentage, and 220 index SNPs for visceral adipose tissue. F-statistics for these genetic instruments were from 23.302 to 1406.670, all larger than the normally selected value of 10 for strong instruments [28]. Details are shown in Table 1 and File S1. For both TH and SA analysis, sensitivity analyses were assessed by IVW and MR-Egger, but most analyses exhibited significant sensitivity (see the results of the heterogeneity test in File S2), so we used the random-effects models in MR analysis. Potential pleiotropy was evaluated via MR-Egger regression intercept and all *p*-values of MR-Egger intercept tests were >0.05, indicating that there was no horizontal pleiotropy. The results are shown in File S2. We estimated the genetic correlation between obesity-related paraments with respect to cerebral imaging-derived phenotypes, such as cortical thickness (TH) and surface area (SA). Global cortical thickness and surface area, as well as their 34 functional regions with global weighting, were included in our comprehensive MR study. Associations of genetically predicted values of these four indexes with the brain-imaging measures are displayed as a heat map in Figure 2. In the primary analysis using IVW, higher BMI was found to be genetically correlated with a thinner global cortical thickness (β: −0.009, SE = 0.003, *p* = 0.006). In the region-based analysis, there were six regions related to BMI, including the fusiform, inferior parietal, pars orbitalis, pars triangularis, supramarginal, and temporal pole. As for the surface area, BMI was found to decrease the SA in the inferior temporal region (β: −17.469, SE = 8.565, *p* = 0.041) but increase the SA in the precuneus (β: 19.397, SE = 9.337, *p* = 0.038) and transverse temporal regions (β: 3.046, SE = 1.540, *p* = 0.048). Body fat percentage, another common measure of obesity, was found to be related to the cortical thickness in several regions, such as the entorhinal, fusiform, inferior parietal, pars orbitalis, and superior parietal, but only to the surface area in the inferior parietal and precentral. It is noted that body fat percentage significantly decreased the thickness of the fusiform (β: −0.014, SE = 0.004, *p* = 9.35 × 10^−4^) and inferior parietal areas (β: −0.014, SE = 0.003, *p* = 9.73 × 10^−6^) after correcting for multiple testing. Similar results to body fat percentage, visceral adipose tissue had a causal relationship with the thickness in the entorhinal, fusiform, and inferior parietal regions, as well as with global thickness. The causal relationship between VAT and global thickness was still significant after multiple-comparison corrections (β: −0.013, SE = 0.004, *p* = 1.34 × 10^−4^). Genetic predicted VAT was associated with the surface area in the isthmus cingulate and precuneus. There was no evidence for a cause–effect of WHR on cerebral cortical thickness or surface area. Details are presented in File S2, and the obesity-related regions are presented in Figure 3.

Because there was no positive result in the two-sample MR of WHR and cerebral cortex phenotype, we only included other obesity indexes (BMI, BFP, and VAT) as the exposures in our subsequent research. In the MVMR, we mutually estimated the effects of BMI, BFP, and VAT on cortical thickness and surface area. After adjusting BMI and VAT, a negative causality of BFP on the thickness of the fusiform gyrus was observed (β: −0.066, SE = 0.030, *p* = 0.029). Similar to the results in two-sample MR analyses, genetically predicted BFP was associated with smaller cortical surface area of the precuneus (β: −226.395, SE = 107.496, *p* = 0.035). Although BMI appeared to be related to the thickness of the superior parietal gyrus in MVMR, the effect of BMI on the global or other six regional cortexes that were significant in MR analyses attenuated to null. Nothing statistically significant was found in the MVMR of VAT (Table 2).

### 3.2. Differential Gene Expression in Brain Regions

**Figure 4:**
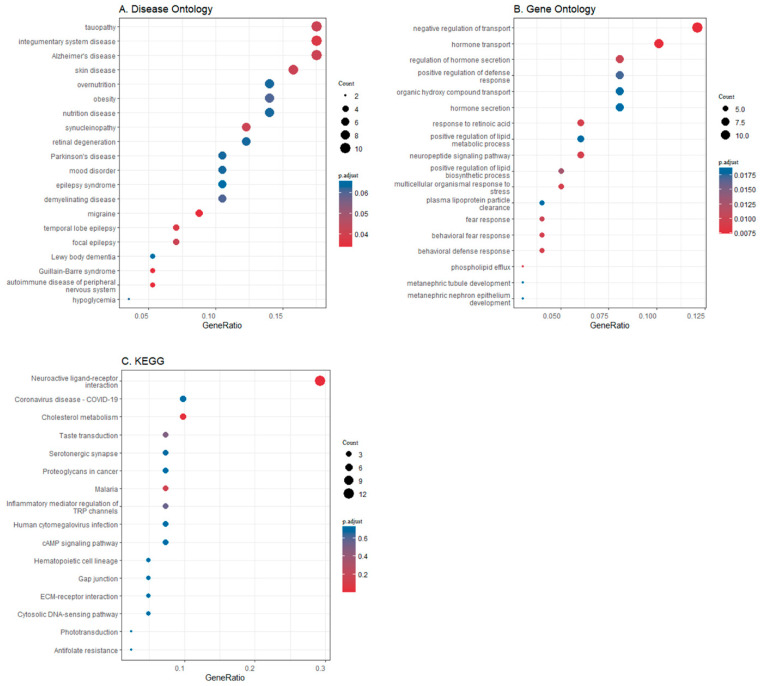
The results of the gene-enrichment analysis. (**A**) Disease ontology (DO); (**B**) Gene Ontology (GO); (**C**) Kyoto Encyclopedia of Genes and Genomes (KEGG).

**Figure 5:**
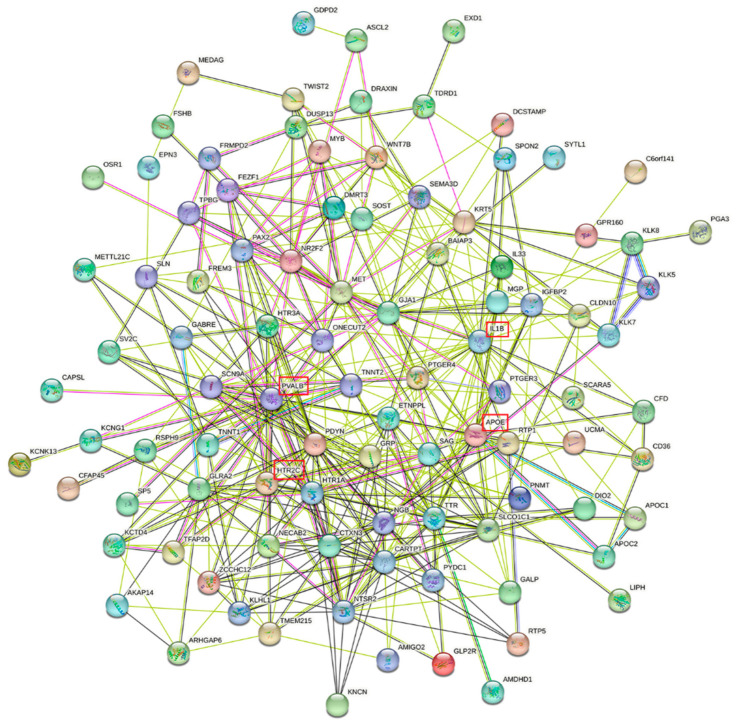
The protein–protein-interaction (PPI) network. The edges indicate functional and physical protein–protein interactions, and the different edge colors represent various evidence of protein connections.

To further integrate obesity, the brain, and genes, we extracted the gene sets from the Allen Human Brain Atlas (AHBA) in the nine brain regions associated with four obesity indexes in the thickness MR study (entorhinal, fusiform, inferior parietal, pars orbitalis, pars triangularis, superior parietal, supramarginal, and temporal pole). Then, all genes expressed in these gyri were transited in entrezid and were enriched in the disease ontology (Figure 4A), the BP terms of GO (Figure 4B), and the KEGG pathway (Figure 4C). Biological processes, such as negative regulation of transport, hormone transport, regulation of hormone secretion, neuropeptide-signaling pathway, and organic hydroxy-compound transport, were examples of significantly enriched GO terms. The gene set was enriched in KEGG pathways such as neuroactive ligand–receptor interaction, cholesterol metabolism, and serotonergic synapse. In the DO-enrichment analysis, these related regions contained a highly enriched set of genes involved in some neuropsychiatric disorders, specifically Alzheimer’s disease, Parkinson’s disease, Lewy-body dementia, tauopathy, migraine, mood disorder, temporal lobe epilepsy, and focal epilepsy. The protein–protein interaction (PPI) network was created by using the website STRING (Figure 5). Network nodes represent proteins, the edges represent functional and physical protein–protein associations, and the line color represents different types of protein-interaction evidence. The green lines represent evidence from text mining, the pink lines evidence from experiments, and the blue lines evidence from databases. Finally, 105 nodes formed a network with 418 edges, and the average node degree was 7.96. Hubs were the most connected nodes within the network and were also responsible for maintaining the connectivity of the network [39]. Some key molecules, such as APOE, IL-1β, HTR2C, PVALB, PDYN, CARTPT, HTR1A, and NR2F2, served as the hub proteins. They formed an interacting community with other proteins. 

We also extracted genes related to the regions associated with obesity indexes in the surface-area MR, but unfortunately, no result was acquired in the enrichment analysis.

## 4. Discussion

Our study was divided into two parts. In the first part, the univariable and multivariable Mendelian randomization study explored potential causal associations between obesity and brain structure. In the second part, we conducted a series of bioinformatics analyses based on the MR results.

Instead of measuring BMI alone to estimate obesity, we added three other fat measures into our research, such as waist-to-hip ratio, body fat percentage, and visceral adipose tissue. Although there were subtle differences in the results of each indicator, there were some common conclusions overall. First, genetically predicted higher levels of BMI or visceral adipose tissue were associated with smaller global cortical thickness. Second, the effects of obesity on the brain were inconsistent across specific brain regions. As for the thickness, the brain gyri affected by obesity included the entorhinal, fusiform, inferior temporal, inferior parietal, pars orbitalis, pars triangularis, superior parietal, supramarginal, and temporal pole. As for the cortical surface area, the affected regions were less than that in the thickness analysis, including the inferior parietal, inferior temporal, isthmus cingulate, and transverse temporal. Only two relationships—the one between BFP and fusiform thickness and the other between BFP and precuneus surface area—survived in the MVMR. This study provides evidence that obesity is associated with a wide range of cortical structural changes.

Inconsistency existed in the results of each indicator. One reason is that the judgment about obesity seems to depend on the anthropometric measure [40]. Not all forms of obesity are equally dangerous, and different indicators suggest various detailed information [41]. BMI, which is calculated as weight in kilograms divided by height in meters squared, is widely used in collecting demographic health information to identify overweight or obesity. However, limitations exist in BMI. Influenced by age, gender, and ethnicity, BMI does not consider the body composition or fat distribution, failing to distinguish between fat, muscle, and bone mass [42]. WHR, the waist-to-hip ratio, provides more information about the abdominal fat distribution, and VAT is associated with fat accumulation in the internal organs. WHR and VAT are closely linked to higher metabolic risks [43]. BFP, the total mass of fat divided by total body mass, focuses on the relative amount of adipose tissue in the whole body instead of the fat distribution [41]. A significant relationship between BFP and the brain structure was found in our univariable and multivariable MR analyses, implying that the amount of adipose tissue is far more meaningful than the simple figure of body weight. In the future, more attention should be paid to the change in BFP in studies about obesity and the brain. 

The association between obesity and the brain remains an ongoing topic. Obese people face higher risks of cognitive impairment and neuropsychiatric disorders [44,45]. In the US, obesity has become the most prominent modifiable risk factor for Alzheimer’s disease and related dementias [46]. Cortical thickness is one of the neuroimaging biomarkers used to assess cognitive-decline risk or disease progression [47]. This study provides evidence supporting the negative association of obesity with global cortical thickness. As for specific brain regions, the effects of obesity are inconsistent. The fusiform gyrus and inferior parietal gyrus were the two main regions. Other gyri were also associated with obesity-related variables, such as the entorhinal, inferior temporal, pars orbitalis, pars triangularis, superior parietal, supramarginal, and temporal pole. Our results are in keeping with a series of previous studies in the literature. In a large-scale study including 6, 420 participants from the ENIGMA MDD working group, Nils Opel reported associations between obesity and lower temporal–frontal cortical thickness, especially in the fusiform gyrus [48]. Among type 2 diabetes patients, global mean cortical thickness was lower in the overweight/obese group than in the normal-weight group, and so was the regional cortical thickness in the fusiform and supramarginal cortex [49]. In another study involving healthy individuals, obese participants showed lower cortical thickness in the pars triangularis, superior frontal gyrus, supramarginal gyrus, inferior parietal cortex, and precuneus [50]. Furthermore, evidence has emerged of the neurological consequences of obesity [51], revealing underlying pathophysiological mechanisms. A study of postmortem brain tissue reported fewer neurons in the overweight/obese subjects even without a cortical-thickness change [52]. Obesity could lead to endothelial dysfunction and inflammation, and these pathological processes accelerate neuronal loss in the brain, which is reflected by decreased gray matter volume [53].

Obesity-related brain changes can explain worse cognitive function among obese individuals [3,53]. The fusiform gyrus and inferior parietal lobe, especially influenced by obesity in our study, are important in cognition and other higher-order brain function [54,55,56]. Obese individuals always show poor performance in cognitive domains, such as memory, attention, verbal fluency, and executive function [53]. Our results can provide solutions for the early identification of cognitive impairment and other neuropsychiatric disorders. 

The cerebral cortical morphometry changes account for some functional alteration or pathogenesis of neuropsychiatric disorders to some extent. The observed regional cortical alterations in obesity showed considerable similarities with corresponding patterns of surface-based morphometry in previously published studies of neuropsychiatric disorders [48]. Therefore, in the second part, we conducted a series of bioinformatics analysis using the data from the AHBA to further test our hypothesis. The GO and KEGG analyses indicated that genes specifically expressed in obesity-related brain regions were involved in some neurophysiological processes, such as compound transport, hormone secretion, immune response, neuropeptide-signaling pathway, and neuroactive ligand–receptor interaction. These results imply potential mechanisms of obesity affecting the brain. In the DO-enrichment analysis, these genes were enriched in cognitive diseases such as Alzheimer’s disease, tauopathy, and Lewy-body dementia. In addition to cognitive decline, these genes were also enriched in other neuropsychiatric disorders, such as Parkinson’s disease, migraines, epilepsy, and mood disorders. 

In the PPI analysis, several hub proteins were identified, including APOE, IL-1β, PVALB and HTR2C. These hubs were related to nervous system or neurological diseases, forming a highly connected interaction network with other genes. The APOE gene product is an apolipoprotein that participates in lipoprotein metabolism. APOE promotes lipid accumulation, and previous studies have demonstrated its association with obesity [57]. APOE4, an isoform of APOE, breaks the balance of deposition and clearance of the amyloid β peptide (Aβ), increases proinflammatory-cytokine production in the brain, and compromises the blood–brain-barrier (BBB) integrity [58]. Thus, APOE4 is considered the strongest genetic risk factor for late-onset sporadic Alzheimer’s disease [59]. IL-1β is a kind of potent proinflammatory cytokine. In neurons, IL-1β activates the MAPK-p38 signaling cascade, stimulating the Aβ synthesis and hyperphosphorylation of tau [60]. The plasma concentration of IL-1β is elevated in obese individuals, and obesity causes cognitive disorders by promoting the inflammation of the central nervous system [61]. Parvalbumin (PVALB) is a protein specifically expressed by GABAergic interneurons, protecting neurons from excess intracellular calcium [62,63]. In line with the ideas of Roberta Magliozzi, the cerebrospinal fluid (CSF) protein level of PVALB could represent a biomarker for cortical damage and cognitive decline [62]. PVALB correlates with obesity as well. Serum levels of PVALB were significantly higher in both obese mice induced by a high-fat diet and obese human individuals [64]. HTR2C, the serotonin 2C receptor, is another protein that is linked to both obesity and advanced brain functions. The loss-of-function variants in the HTR2C gene (encoding the HT2CR protein) could lead to the development of obesity in humans [65]. Inactivation of HT2CR in the ventral hippocampus impairs behavioral performance in a visual-detection task that demands attention [66].

## 5. Limitations and Future Research

Combing MR analysis and bioinformatics analysis, our study illustrates how obesity is linked to the human brain from the perspectives of genes, structure, and disease. All of the above results not only further enrich the conclusions of our study, but also verify the hypothesis that obesity affects the structure and function of the brain, thus leading to neuropsychiatric disease. However, this study also has limitations that need to be acknowledged. First, the available data in our MR analysis were based on individuals of European ancestry, so the causal relationship in other ethnicities remains unknown. Second, the cortical effects of obesity may be mediated by age or sex [48], but the role of sex and age was not considered in our study as a result of the lack of data stratified by age and sex. Third, our study only estimated the mean of both hemispheres. The hemisphere-specific (left or right) effect of obesity raises the need for further investigation. Fourth, the genetic information from the AHBA was limited to a small sample size: six donors of different ages and sex in the US [37]. Last, the messages about obesity-related brain structures in this study should be interpreted with caution. Brain-structural and gene-expression changes related to obesity cannot be concluded from the current study design. Our results provide only a hypothesis for the relationship between obesity and the brain. Therefore, further studies are needed in the future.

Finally, some problems deserve to be noted. Biological-sex differences are displayed in the human brain, involving cognition, behavior, and regional brain anatomy [67]. One limitation of our implementation is that we did not perform further analyses by age or sex. It remains unclear to which degree gender or age is attributed to structural changes in the cerebral cortex. Future research should aim to investigate whether either age or gender moderates or mediates the connection between obesity and the brain. In addition, obesity is often accompanied by biochemical-indicator disturbance and metabolic changes. Some biochemical indicators are strongly associated with obesity, such as serum leptin, glucose, and insulin, alongside pro-inflammatory cytokines and C-reactive protein [68]. These biochemical markers have been widely considered in studies aimed at understanding the complex interplay between obesity and the brain [69]. However, there is still no MR study confirming the role of these biochemical indicators. Clinical biochemical findings provide valuable insights into the physiological and metabolic changes, which are fundamental for exploring potential mechanisms underlying the effects of obesity on brain function and structure. 

## 6. Conclusions

In the present study, we provide genetic evidence of the causal association between obesity and cortical morphometry at the global and regional levels. The brain region-specific alterations affected by obesity are also linked to neurological diseases such as Alzheimer’s disease and other neurodegenerative diseases. Clarifying the link between obesity, brain structure, and disease may offer new avenues for the treatment of cognitive degeneration. These results emphasize the importance of proper body weight for a healthy brain.

## Figures and Tables

**Table 1:** Summary of exposure factors.

Exposure	Unit	Consortium or Study	Sex	Sample Size	Population	SNP Number	*F*-Value
**BMI**	SD (kg/m^2^)	GIANT+UKB	Males and females	681,275	European	513	74.036
**WHR**	SD	GIANT+UKB	Males and females	697,734	European	72	71.708
**BFP**	SD (%)	UKB	Males and females	492,787	European	300	42.851
**VAT**	SD (kg)	UKB	Males and females	325,153	European	220	57.661

GIANT: Genetic Investigation of ANthropometric Traits (GIANT) consortium; UKB: UK Biobank; BMI: body mass index; WHR: waist-to-hip ratio; BFP: body fat percentage; VAT: visceral adipose tissue.

**Table 2:** Causal relationships of obesity indexes and brain-structure phenotypes estimated by multivariable MR.

Exposure	NSNP	Beta	SE	*p*	Exposure	NSNP	Beta	SE	*p*
CT−global					SA_inferior parietal				
BMI	20	−0.026	0.052	0.623	BMI	20	268.238	241.654	0.267
BFP	20	0.006	0.033	0.860	BFP	20	13.166	156.641	0.933
VAT	21	0.015	0.055	0.787	VAT	21	−315.274	255.332	0.217
CT_entorhinal					SA_inferior temporal				
BMI	20	0.084	0.163	0.607	BMI	20	−167.396	139.979	0.232
BFP	20	−0.032	0.106	0.762	BFP	20	−148.798	90.549	0.100
VAT	21	−0.023	0.173	0.894	VAT	21	214.826	147.914	0.146
CT_fusiform					SA_isthmus cingulate				
BMI	20	−0.007	0.046	0.875	BMI	20	−25.197	62.640	0.688
BFP	20	−0.066	0.030	0.029 *	BFP	20	−43.801	40.681	0.282
VAT	21	0.031	0.048	0.519	VAT	21	73.874	66.186	0.264
CT_inferior parietal					SA_precentral				
BMI	20	−0.016	0.038	0.676	BMI	20	23.006	152.876	0.880
BFP	20	0.020	0.024	0.399	BFP	20	−71.641	98.818	0.468
VAT	21	0.007	0.040	0.852	VAT	21	−10.743	161.507	0.947
CT_inferior temporal					SA_precuneus				
BMI	20	0.058	0.047	0.215	BMI	20	−92.171	166.077	0.579
BFP	20	0.036	0.030	0.238	BFP	20	−226.395	107.496	0.035*
VAT	21	−0.083	0.049	0.091	VAT	21	240.129	175.493	0.171
CT_pars orbitalis					SA_transverse temporal				
BMI	20	−0.118	0.067	0.079	BMI	20	18.932	27.781	0.496
BFP	20	0.005	0.044	0.913	BFP	20	14.190	18.018	0.431
VAT	21	0.105	0.071	0.141	VAT	21	−11.132	29.354	0.705
CT_pars triangularis									
BMI	20	−0.059	0.068	0.383					
BFP	20	−0.060	0.043	0.165					
VAT	21	0.079	0.071	0.268					
CT_superiorparietal									
BMI	20	−0.090	0.044	0.038 *					
BFP	20	0.026	0.028	0.351					
VAT	21	0.077	0.046	0.095					
CT_supramarginal									
BMI	20	−0.014	0.046	0.764					
BFP	20	0.016	0.030	0.590					
VAT	21	−0.003	0.048	0.953					
CT_temporalpole									
BMI	20	0.026	0.147	0.860					
BFP	20	−0.063	0.095	0.509					
VAT	21	−0.003	0.155	0.983					

Abbreviations: BMI: body mass index; BFP: body fat percentage; VAT: visceral adipose tissue; CT: cortical thickness; SA: surface area; NSNP: number of SNPs used in MR; beta: β, estimate of the effect; SE: standard error; * *p*-value < 0.05.

## Data Availability

Publicly available datasets were analyzed in this study. This data can be found here. The summary statistics of GWASs for body mass index (BMI) and waist-to-hip ratio (WHR) were derived from the GIANT Consortium page, https://portals.broadinstitute.org/collaboration/giant/index.php/GIANT_consortium_data_files (accessed on 20 October 2022). Summary statistics for body fat percentage (BFP) are available from the UK Biobank website, http://www.nealelab.is/uk-biobank, accessed on 20 October 2022. Summary statistics for visceral adipose tissue (VAT) can be accessed from a GWAS conducted by Torgny Karlsson et al. (DOI: 10.1038/s41591-019-0563-7) at https://myfiles.uu.se/ssf/s/readFile/share/3993/1270878243748486898/publicLink/GWAS_summary_stats_ratios.zip (accessed on 29 October 2022). The datasets of the human cerebral cortex can be downloaded from the ENIGMA Consortium website, but restrictions apply to the availability of these data, which were used under license for the current study. Data are available from the authors upon reasonable request and with the permission of ENIGMA—Genetics working group (DOI: 10.1126/science.aay6690). The spatial-transcriptome data were selected from the Allen Human Brain Atlas (AHBA), https://human.brain-map.org/ (accessed on 30 December 2022).

## Supplementary Materials

The following supporting information can be downloaded at: https://www.mdpi.com/article/10.3390/brainsci13060892/s1, File S1: SNPs for Mendelian randomization. Table S1–S4. List of the selected SNPs for each measure of obesity. File S2: Results of the brain cortical thickness and surface area. Table S5. Results for the Mendelian-randomization estimates and sensitivity analysis in cortical thickness analysis. Table S6. Results of the Mendelian-randomization estimates and sensitivity analysis in cortical surface area analysis. File S3: Figure S1. The results of GO analysis (CC: cellular components). Figure S2. The results of GO analysis (MF: molecular functions).

## Abbreviations

AHBA	Allen Human Brain Atlas
BFP	body fat percentage
BMI	body fat index
BP	biological processes
CT	cortical thickness
DO	Disease Ontology
ENIGMA	Enhancing NeuroImaging Genetics through Meta-Analysis
GIANT	Genetic Investigation of ANthropometric Traits
GO	Gene Ontology
GWAS	genome-wide association study
IVW	inverse-variance weighting
KEGG	Kyoto Encyclopedia of Genes and Genomes
MR	Mendelian randomization
MRI	magnetic resonance imaging
PPI	protein–protein interaction
SA	surface area
SNPs	single nucleotide polymorphisms
WHR	waist-to-hip ratio
VAT	visceral adipose tissue
