# Supplementary material for: Genetic Insights into Obesity and Brain: Combine Mendelian Randomization Study and Gene Expression Analysis
Source: Brain Sci. 2023 May 31;13(6):892. doi: 10.3390/brainsci13060892 (PMC10295948; doi:10.3390/brainsci13060892)
Supplement: Supplementary file 1 [file brainsci-13-00892-s001.zip › Supplementary material/supplementary 3.docx]

Supplementary material

Figure S1. The results of GO analysis (CC).


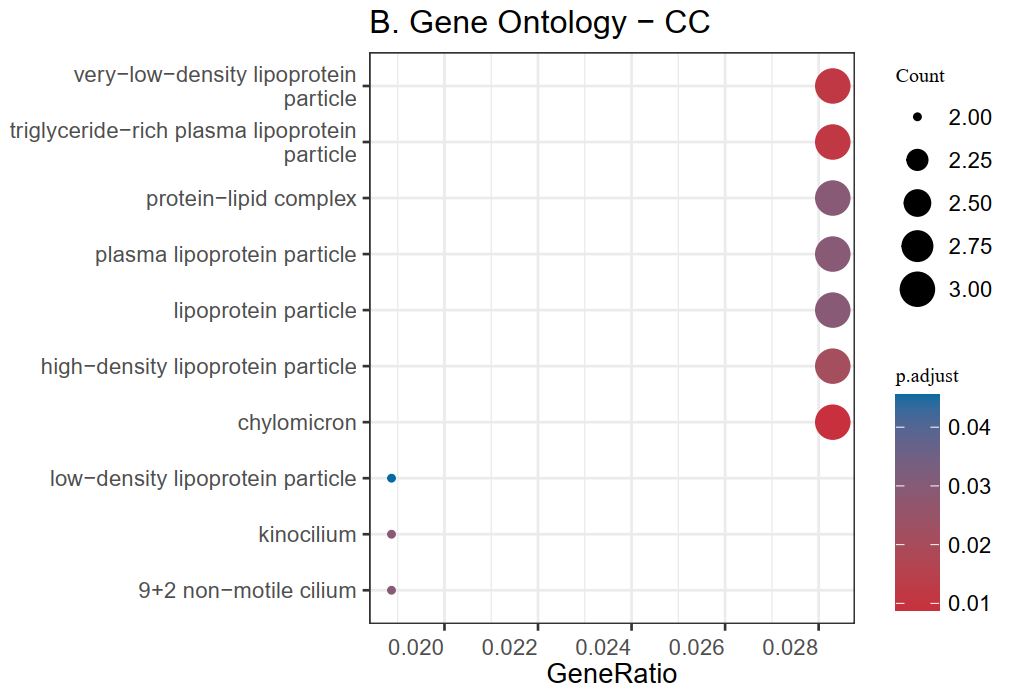


GO- Gene Ontology;

CC- cellular component

Figure S2. The results of GO analysis (MF).


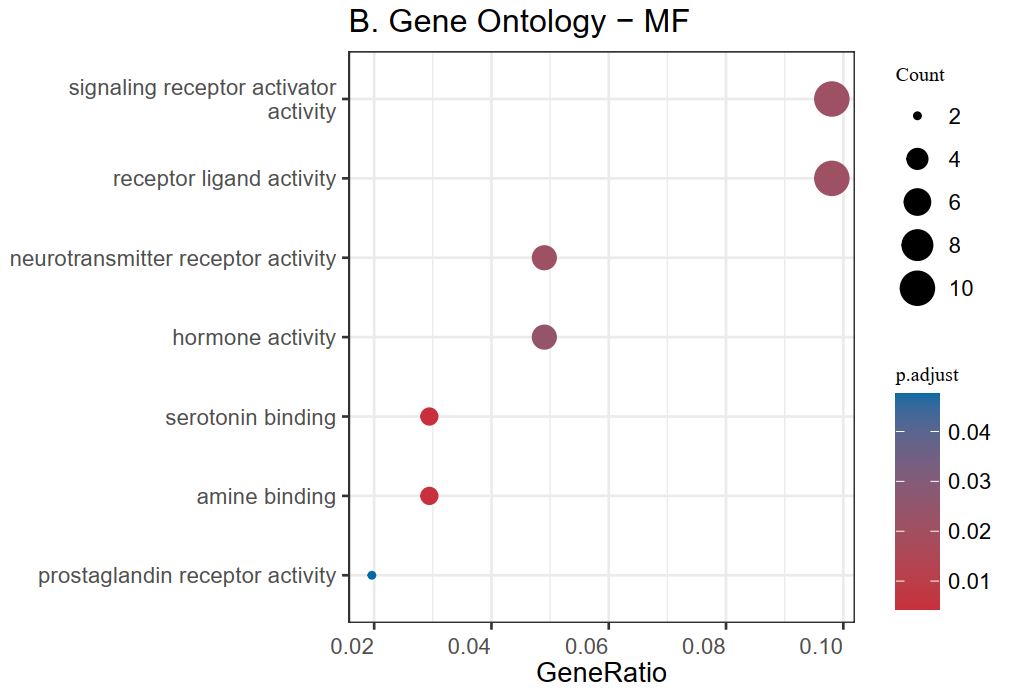


GO- Gene Ontology;

MF- molecular function
